# The Case for the Tuberculous Child

**Published:** 1908-06-06

**Authors:** 


### The Hospital

A Journal of

The Medical Sciences and Hospital Administration.

New Series. No. 64. Vol. III. [No. 1136, Vol. XLIV.] SATURDAY, JUNE 6, 1908.

### THE CASE FOR THE TUBERCULOUS CHILD.

During the last decade our views on tuberculosis and its treatment have undergone important modifications with far reaching results. The fight for the admission of the fact that tubercle is a curable lesion, due to a specific cause, begun by Laennec almost a century ago, reached victorious culmination with the demonstration of the specific bacillus and the excellent and encouraging results obtained by various methods of treatment. To-day the rôles of prophylaxis and sanatoria in the war against tuberculosis are universally considered as of primary importance. Almost in every country the State has taken upon itself to make regulations which have for their object the prevention of the further spread of the disease. Almost every district in this country has a sanatorium for the treatment of consumptive patients, and one of the most significant and most encouraging signs of our advance towards the final goal is the manner in which the nation is awakening to a sense of its responsibilities in this great subject, so far as the infant population is concerned.

Up to comparatively a few years ago the case of the tuberculous child was one which hardly received the attention it warranted. It needs only a casual glance at statistics to see how imperatively necessary it was to deal with it. The death-rate among children from tuberculosis has been steadily rising in Germany, America, and Russia. In this country it is difficult to obtain definite statistics on this point, but no one who is acquainted with our large children's hospitals, and, for that matter, with the out-patient departments at all general hospitals, can for a moment doubt that here, too, there has been an equally certain increase in the mortality. Statistics, it is true, count for little, and we do not wish to press them, for the obvious facts of the case are known to all our readers. Who has not seen, with a despairing realisation of his own impotence adequately to deal with such cases, the "recurrent tuberculous patients" that crowd our out-patient departments, and that prove so hopelessly unsatisfactory in private practice? Who, again, has not witnessed the equally sad cases which are treated as in-patients, time after time, to come finally to the post-mortem room or to go through life as broken men and women, with their economic value to the community enormously lowered? The tuberculous hip comes into a ward where he or she is given the best treatment that medical or surgical art can give. The case leaves the wards "improved," and the first stages of cure are accomplished at the convalescent home. The patient goes back to his slum, with its squalid surroundings and unhygienic environment, with the inevitable result that he breaks down again. Such cases are the despair of the surgeon and a standing menace to the health of the community; yet hitherto they have received far less attention than adult cases, in which the need for prompt and united action is less evident. For these reasons it is satisfactory to find that there is a movement to take up the case of the tuberculous child and to give it at least the same consideration that has been accorded to the case of the tuberculous adult. The Stony Wold Sanatorium, planned and completed four years ago through the energy and devotion of two New York doctors, has initiated the era of the systematic sanatorium treatment of tuberculous children, and its undoubted success should be an encouragement for others to effect in this country what has been so excellently achieved in America. In Germany two children's sanatoria are in being, both small, it is true, but both doing good work. In England two children's "consumption homes" are in course of construction, and since 1906 the Holt Children's Sanatorium has been receiving, as a partially endowed institution, as many cases as its limited means allowed. It is not, however, to the sanatorium that we must look as an adequate remedy for the elimination of infantile tuberculosis. Rather is it to hygienic measures, prophylactic attempts, and the provision of better homes and better local conditions for the mass of our slum children from whose ranks by far the greater number of tuberculous patients is drawn. France, a country which has been well in the front rank in dealing with the disease among her children, has with praiseworthy foresight made this latter method one of the main, if not the main, feature of her programme, and Germany has rapidly followed on much the same lines—lines which are worthy of imitation. The establishment of open-air "colonies" for strumous children is an important point to consider. Where such colonies have been working they have proved highly satisfactory, as at Charlottenburg, where a Forest School exists, and also at the various tuberculous "circles" in France whereat more than 6,000 children are treated annually. Another scheme, which has so far met with but moderate success, has been the "boarding-out system" in which slum children are given the chance of spending at least a few months every year amidst country surroundings under far better hygienic and dietetic conditions than obtain in their own homes. All these measures have met with encouraging success, and the great drawback to them all, the question of initial expense, has been amply compensated by the undoubted advantages that have accrued from them. Here in England they demand, at least, a fair trial. The question of expense is one which should concern the ratepayer and which should not be left to the charity of private societies or individuals. The case for the tuberculous child is a case for national consideration and should be treated as such. It is only by prompt effort that it can be bettered, and the welfare of the whole community, in each of our large cities, demands imperatively that it should be bettered without delay.

